# Discrete Modeling of Amoeboid Locomotion and Chemotaxis in *Dictyostelium discoideum* by Tracking Pseudopodium Growth Direction

**DOI:** 10.1038/s41598-017-12656-1

**Published:** 2017-10-04

**Authors:** Zahra Eidi

**Affiliations:** Department of Physics, Institute for Advanced Studies in Basic Sciences (IASBS), Zanjan, 45137-66731 Iran

## Abstract

*Dictyostelium discoideum* amoeba is a well-established model organism for studying the crawling locomotion of eukaryotic cells. These amoebae extend pseudopodium - a temporary actin-based protrusion of their body membrane to probe the medium and crawl through it. Experiments show highly-ordered patterns in the growth direction of these pseudopodia, which results in persistence cell motility. Here, we propose a discrete model for studying and investigating the cell locomotion based on the experimental evidences. According to our model, *Dictyostelium* selects its pseudopodium growth direction based on a second-order Markov chain process, in the absence of external cues. Consequently, compared to a random walk process, our model indicates stronger growth in the mean-square displacement of cells, which is consistent with empirical findings. In the presence of external chemical stimulants, cells tend to align with the gradient of chemoattractant molecules. To quantify this tendency, we define a coupling coefficient between the pseudopodium extension direction and the gradient of an external stimulant, which depends on the local stimulant concentration and its gradient. Additionally, we generalize the model to weak-coupling regime by utilizing perturbation methods.

## Introduction

Amoeboid movement is the most common method of locomotion in eukaryotic cells^1^. This structured movement is widely seen in unicellular organisms with amorphous structures, *e*.*g*. in leukocyte crawling within interstitial tissues^2^. A model organism for eukaryotic cellular motility research is *Dictyostelium discoideum*. *Dictyostelium* is a free-living soil amoeba, feeding on bacteria. When the nutrients are available, *Dictyostelium* lives as a single-cell amoeba with nearly round spherical shape with average diameter of 10 *μm*. When the food runs out, as a survival strategy, the cells start to signal by releasing cyclic Adenosine Mono Phosphate (cAMP) in to the environment to attract other cells^3^. The nearby cells respond to this signal both by relaying the signal and moving up the cAMP gradient. This directed movement in response to extracellular chemical stimulants is called Chemotaxis. The chemotaxis process prompt self-accelerating processes results in aggregation^4–6^. The aggregation process leads to form a multicellular organism whose shape evolves in time. Finally, the process forms a structure consists of a stalk and a fruiting body including spores which are capable of long-term survival.

In order to probe the medium and move in the environment, the amoeba cells grow pseudopodium, a temporary actin-based protrusion of their membrane. Based on the experiments the cell extends pseudopodia in two types: (1) splitting on certain angles with respect to the existing pseudopodium, (2) growing protrusions occasionally at the rear side of the cell, called de novo^7^. The amoeba is propelled by growing successively splitting pseudopodia in a particular direction, while a random reorientation is generated by extending de novo pseudopodium^8–10^. Before starved cells sense their neighbors and begin to cooperate with them, the locomotion of the cells can be modeled as persistent random walk^9–13^. It is assumed that the mechanism of persistent movement in *Dictyostelium discoideum* depends on the ratio of splitting and de novo pseudopodia. External chemical stimulants may bias the position and direction of pseudopod extension. For example, in the presence of cAMP concentration the cells tend to align their movement with the stimulant gradient^10^.

Having a theoretical comprehension of the cell’s directed random walk is of high importance from phenomenological point of view. The mathematical modelling of cell movement goes back to Patlak^14^ (1950*s*), E. Keller and L. Segel^15^ (1970*s*). The model is composed of a set of coupled differential equations for density of cells and concentration of chemoattractants. The study of the cells migration statistics via pursuing the tracers has been developed in recent years. The studies cover experimental and theoretical modeling of cell movement both in the absence and presence of external chemoattractants. The experiments include tracking the cell’s centroid^11–13^ or pseudopodia growth direction^9,10^. From theoretical point of view there are two main approaches to deal with this subject: writing a Langevin equation for the cell’s velocity vector which is represented by an angle and a modulus and writing a Fokker-Planck equation for the cell’s position. In the homogenous medium most of these works^9–13,16–18^ share this consequence that the cells go through persistent movement on short time scales and an ordinary diffusive behavior on long time scales. However, complex dynamics with anomalous diffusion has been also reported for the spontaneous movement of *Dictyostelium discoideum* ^19,20^. Same anomalies were also observed in the mean square displacement of wild-type epithelial canine kidney cells^21^ as well as Hydra cells^22^. The experiments also show that speed control and reorientation cycles of the cell are synchronized and negatively correlated^23^. Recently, more complex models of *Dictyostelium* trajectories, quantifying the persistence degree in random amoeboid motion based on Hurst exponent of Brownian motion has been proposed^24^.

In an inhomogeneous medium, although it is the gradient of cAMP that conducts the movement of the individuals (Chemotaxis), the absolute value of cAMP concentration also plays a key role (Chemokinesis). Indeed, the cell’s motility depends on some presently-unknown combination of local cAMP concentration and its steepness. There are evidences that indicates the cells undergo a motion with multiplicative noise^18^. Chemotactic motion of these cells is the subject of research of variant insights ranging from corresponding biochemical inter-cellular pathways^25^ to cell-substrate adhesive drag forces^26^.

The aim of the present work is to make a simple discrete model based on the experimental observations for *Dictyostelium* migration, first in a uniform environment and then in the presence of external signaling. A few points regarding the wild type of cell movements are observed^9^:

1. Pseudopodia are extended perpendicular to the surface curvature at the place where they emerge^27^.
2. Two types of pseudopodia may be formed: frequent splitting of an existing pseudopod, or the occasional extension of a de novo pseudopod at regions devoid of recent pseudopod activity^7^.
3. The angle between two split-split pseudopodia is bimodally distributed with peaks of about 55 degrees to the right or left relative to the previous pseudopod.
4. De novo pseudopodia are extended with equal probability in nearly random directions.
5. A pseudopod can extend to the right (**R**, positive angle) or to the left (**L**, negative angle) relative to the previous pseudopod. The alternating **RL** + **LR** occur about 3 times more often than the consecutive **RR** + **LL**.
6. The pseudopodia do not bend towards the gradient and still are extended perpendicular to the local cell surface curvature^10^.

Here, considering the above observations as axioms of the macroscopic dynamics of the cell locomotion, we propose a stochastic model for the movement. We describe the model as a “second order Markov chain” for the direction of movement, meaning that the subsequent direction depends not only on the present direction, as in a standard Markov process, but also on the previous direction. Coupling the centroid’s movement to the ordered pseudopod growth process, we show that in the absence of external signaling the model leads to undirected motion. Afterwards, by combining the rules of cell’s motility with its inclination towards the gradient of external stimulants concentration, we see that the model result in biased movement. The results matches fairly well with the corresponding experiments. This method might help shed light on the question that what are the functional quantities in collective behavior of cells which undergo chemotaxis during the aggregation process.

## Methods

**Figure 1:**
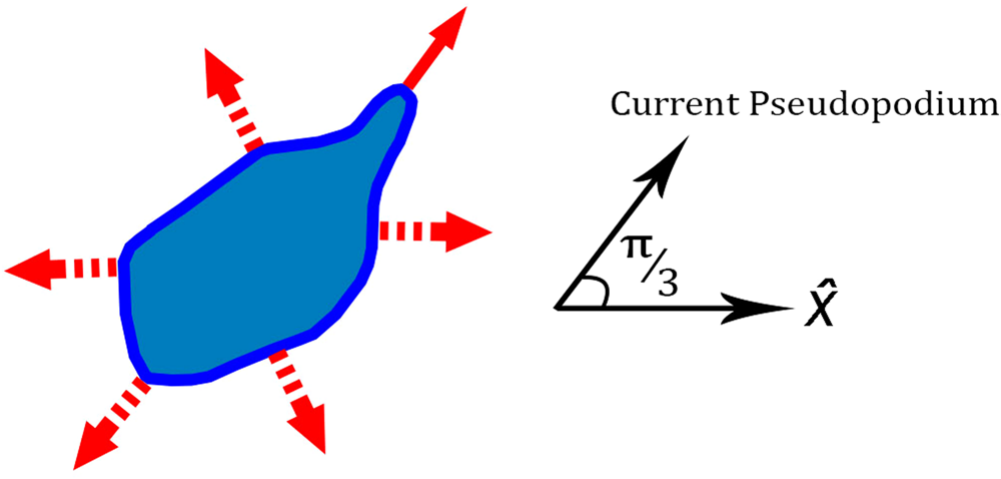
Schematics of the space of states including different possibilities for extending a pseudopodium relative to a given axis, $\hat{x}$. Solid arrow shows the cell’s decision among all other possible alternatives which are shown in dash arrows.

It is a widely held view that the mechanism of persistent movement in *Dictyostelium discoideum* likely depends on pseudopodia extension series. We are about to construct a minimal model based on the aforementioned axioms that would be capable of explaining the experimental data. Let us suppose that a pseudopodium can extend only along six equally divided allowed directions with respect to a fixed axis (see Fig. 1). Thus, the space of states is equal to

1 $$S=\{\frac{(n-\mathrm{1)}\pi }{3}|n\in \{1,2,3,4,5,6\}\}$$

In the following, we imitate the cell movement both in homogeneous and inhomogeneous medium by referring to these directions.

### Movement in Homogeneous Medium

Based on the number 5 of aforementioned observation, indicated in the introduction, there are some statistical correlations between the growth direction of two successive pseudopodia. Indeed, the probability of splitting a pseudopodium in the same direction (turning to left or right) as the previous pseudopodium differs from the probability for a step back. In this case, the stochastic variable of pseudopodium growth direction, *θ*_*t*_, is not a Markov chain but a Markovian of second degree. That is because its probability distribution at time *t* not only depends on its value at *t* − 1 but also depends on its value at *t* − 2. However, by applying a trick the stochastic variable can reduce to a Markov chain. One can test that the pair of consequent growing pseudopods *X*_*t*_ = (*θ*_*t*_, *θ*_*t*−1_) is Markovian^28^. We assume that growing pseudopodia in a row is not allowed. Thus, the range of *X*_*t*_ is a discrete set that consists of a 30 possible states of allowed pair angles and the transition probability *P* ^(0)^(*X*_*t*+1_|*X*_*t*_) is a 30 × 30 matrix.

The transition matrix reads as

2 $$\begin{array}{rcl}{P}^{\mathrm{(0)}}({X}_{t+1}|{X}_{t}) & = & {P}^{\mathrm{(0)}}(({n}_{t+1},{m}_{t+1})|({n}_{t},{m}_{t}))\end{array}$$

3 $$\begin{array}{rcl}\quad \quad \quad \quad \quad \,\, & = & {P}^{\mathrm{(0)}}(({n}_{2},{m}_{2})|({n}_{1},{m}_{1})),\end{array}$$

in which the angle of first and second component of each pairs are indicated by *n* and *m* (*n*, *m* $\in$ *S*), respectively. Note that *m*_*t*+1_ = *n*_*t*_, as both of them address to *θ*_*t*_. We assume that the process is time-homogeneous. Thus the equality in Eq. 3, is straightforward. The zero superscript in *P* ^(0)^ denotes the transition probabilities in the absence of the chemoattractant concentration gradient. Now, after restoring the Markov character, the model can be treated normally by defining the transition probabilities between different states.

Let us assume that in every time step there is only one growing pseudopodium. The pseudopodium growth direction in every time step is a stochastic variable, *θ*_*t*_ $\in$ *S*. The angle *θ*_*t*+1_ on the next step will be in one of the below forms with respect to *θ*_*t*_ (see Fig. 1):

- makes a turn of *π*/3 to the right in the currently active front area of the cell (splitting to the right),
- makes a turn of *π*/3 to the left in the currently active front area of the cell (splitting to the left),
- makes a turn of +2*π*/3, −2*π*/3 or *π* in the currently inactive rear side of the cell (De novo).

**Figure 2:**
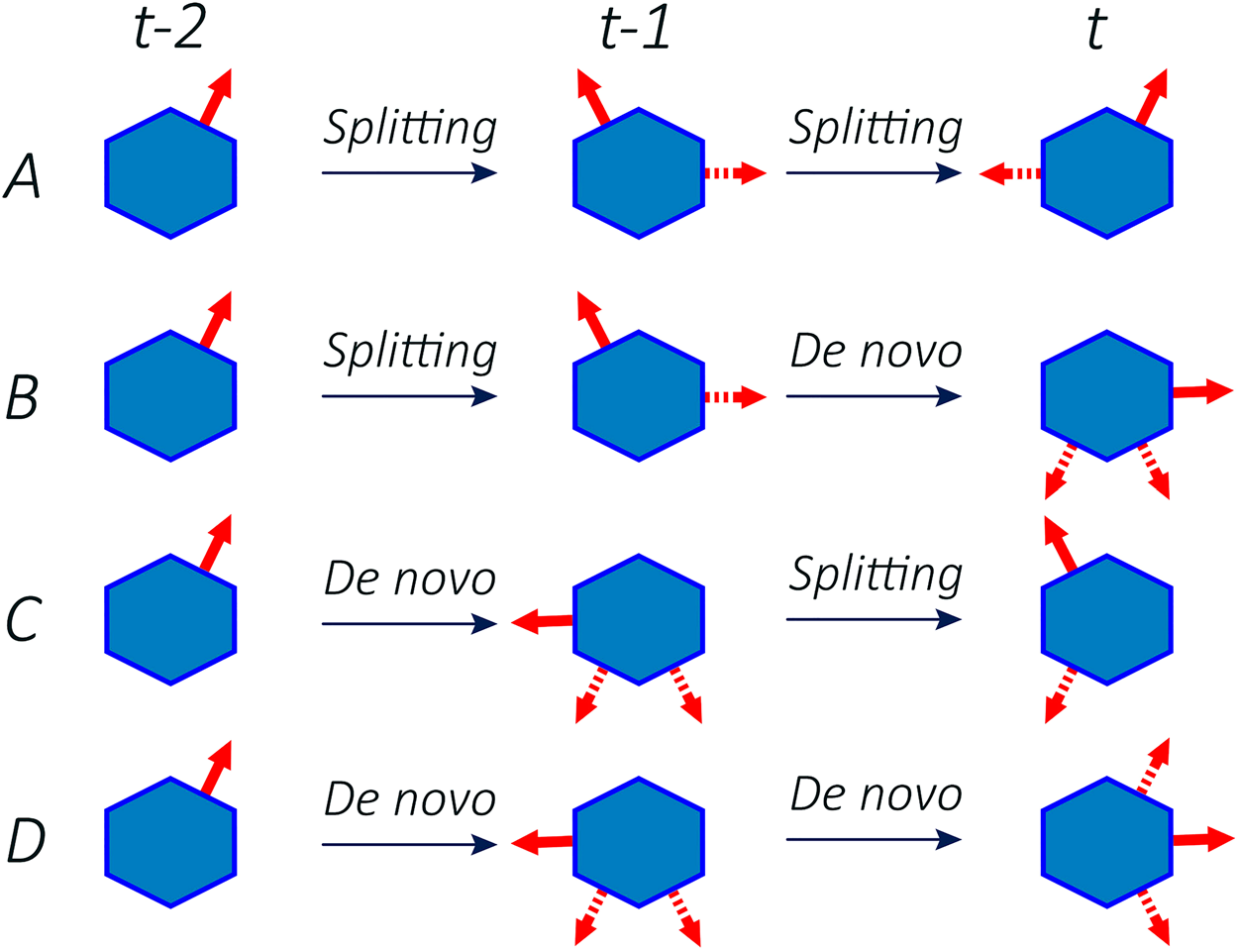
Schematics of the model, showing different possibilities for two successive movement (**A**) splitting-splitting, (**B**) splitting- de novo, (**C**) de novo- splitting, and (**D**) de novo- de novo. Solid arrow shows the cell’s decision among all other possible alternatives which are shown in dash arrows.

There are four scenarios for proceeding every two subsequent steps, See Fig. 2. Every scenario represents the corresponding entries in the transition matrix *P* ^(0)^(*X*_*t*+1_|*X*_*t*_):

(A) Two subsequent splittings, Fig. 2A.
  4 $$\begin{array}{rcl}{P}^{\mathrm{(0)}}(({n}_{2},{m}_{2})|({n}_{1},{m}_{1})) & = & \mathrm{(1}-p){\delta }_{{m}_{2},{n}_{1}}\{{\delta }_{{n}_{2},{n}_{1}+1}(\alpha \,{\delta }_{{n}_{1},{m}_{1}+1}+\beta \,{\delta }_{{n}_{1},{m}_{1}-1})\\  &  & +{\delta }_{{n}_{2},{n}_{1}-1}(\beta \,{\delta }_{{n}_{1},{m}_{1}+1}+\alpha \,{\delta }_{{n}_{1},{m}_{1}-1})\}\end{array}$$
  where *p* is the probability of making a de novo pseudopodium in each step and *α* and *β* are the probabilities of consecutive and alternative splitting, respectively.
(B) Splitting at the side of previous pseudopod at first step and then growing protrusion at the rear side at the second step, Fig. 2B.
  5 $$\begin{array}{rcl}{P}^{\mathrm{(0)}}(({n}_{2},{m}_{2})|({n}_{1},{m}_{1})) & = & \frac{p}{3}{\delta }_{{m}_{2},{n}_{1}}\{{\delta }_{{n}_{1},{m}_{1}+1}\mathrm{(1}-{\delta }_{{n}_{2},{n}_{1}}\mathrm{)(1}-{\delta }_{{n}_{2},{n}_{1}+1}\mathrm{)(1}-{\delta }_{{n}_{2},{n}_{1}-1})\\  &  & +{\delta }_{{n}_{1},{m}_{1}-1}\mathrm{(1}-{\delta }_{{n}_{2},{n}_{1}}\mathrm{)(1}-{\delta }_{{n}_{2},{n}_{1}+1}\mathrm{)(1}-{\delta }_{{n}_{2},{n}_{1}-1})\}\end{array}$$
(C) Extending pseudopodium at the rear side (with respect to the position of previous pseudopodium) and then splitting at the side of the current one, Fig. 2C.
  6 $$\begin{array}{rcl}{P}^{\mathrm{(0)}}(({n}_{2},{m}_{2})|({n}_{1},{m}_{1})) & = & \frac{1-p}{2}{\delta }_{{m}_{2},{n}_{1}}\{{\delta }_{{n}_{2},{n}_{1}+1}\mathrm{(1}-{\delta }_{{n}_{1},{m}_{1}}\mathrm{)(1}-{\delta }_{{n}_{1},{m}_{1}+1}\mathrm{)(1}-{\delta }_{{n}_{1},{m}_{1}-1})\\  &  & +{\delta }_{{n}_{2},{n}_{1}-1}\mathrm{(1}-{\delta }_{{n}_{1},{m}_{1}}\mathrm{)(1}-{\delta }_{{n}_{1},{m}_{1}+1}\mathrm{)(1}-{\delta }_{{n}_{1},{m}_{1}-1})\}\end{array}$$
(D) Growing pseudopod on both steps randomly, at least with 2*π*/3 deviation clockwise or counter-clockwise, with respect to the previous step, Fig. 2D.

7 $$\begin{array}{rcl}{P}^{\mathrm{(0)}}(({n}_{2},{m}_{2})|({n}_{1},{m}_{1})) & = & \frac{p}{3}{\delta }_{{m}_{2},{n}_{1}}\{\mathrm{(1}-{\delta }_{{n}_{1},{m}_{1}-1}\mathrm{)(1}-{\delta }_{{n}_{1},{m}_{1}}\mathrm{)(1}-{\delta }_{{n}_{1},{m}_{1}+1})\\  &  & +\mathrm{(1}-{\delta }_{{n}_{2},{n}_{1}}\mathrm{)(1}-{\delta }_{{n}_{2},{n}_{1}+1}\mathrm{)(1}-{\delta }_{{n}_{2},{n}_{1}-1})\}\end{array}$$

The entire transition matrix is demonstrated in Supporting Information, Appendix A. In general, there are five nonzero entries in each row. Thus, the matrix *P* ^(0)^ is sparse.

In the transition matrix *P* ^(0)^ (see Supporting Information, Appendix A for details) all the entries are non-negative and each row adds up to unity. Clearly, the matrix *P* ^(0)^ has the eigenvalue 1, because ${\sum }_{j=1}^{30}\,{P}_{i,j}^{\mathrm{(0)}}=1$. This relation in matrix notation can be written as *P* ^(0)^ **1** = **1**, where **1** is a column vector whose entries are all 1. *P* ^(0)^ has also a left eigenvector *ω*_0_ corresponding to eigenvalue 1. *ω*_0_ is called a stationary or invariant distribution and can be obtained as

8 $${\omega }_{0}({\mathbb{I}}-{P}^{\mathrm{(0)}})=0.$$

where ${\mathbb{I}}$ is the unite matrix of size 30. We solve Eq. 8, to find *ω*_0_ as a 30 × 1 vector whose entries are invariant probabilities of every possible pairs of subsequent pseudopodia directions. Then, we rearrange the entries in a 6 × 6 array, in which both horizontal and vertical values are in the space of states. Clearly, in the new arrangement all the diagonal entries are zero, as we assumed that growing pseudopodia in a row is prohibited. *ω*_0_ is a symmetric matrix as it is the invariant distribution of extending pseudopodium along discrete directions in a homogeneous medium.

$${\omega }_{0}=(\begin{array}{cccccc}0 & \frac{1-p}{12} & \frac{p}{18} & \frac{p}{18} & \frac{p}{18} & \frac{1-p}{12}\\ \frac{1-p}{12} & 0 & \frac{1-p}{12} & \frac{p}{18} & \frac{p}{18} & \frac{p}{18}\\ \frac{p}{18} & \frac{1-p}{12} & 0 & \frac{1-p}{12} & \frac{p}{18} & \frac{p}{18}\\ \frac{p}{18} & \frac{p}{18} & \frac{1-p}{12} & 0 & \frac{1-p}{12} & \frac{p}{18}\\ \frac{p}{18} & \frac{p}{18} & \frac{p}{18} & \frac{1-p}{12} & 0 & \frac{1-p}{12}\\ \frac{1-p}{12} & \frac{p}{18} & \frac{p}{18} & \frac{p}{18} & \frac{1-p}{12} & 0\end{array})$$

**Figure 3:**
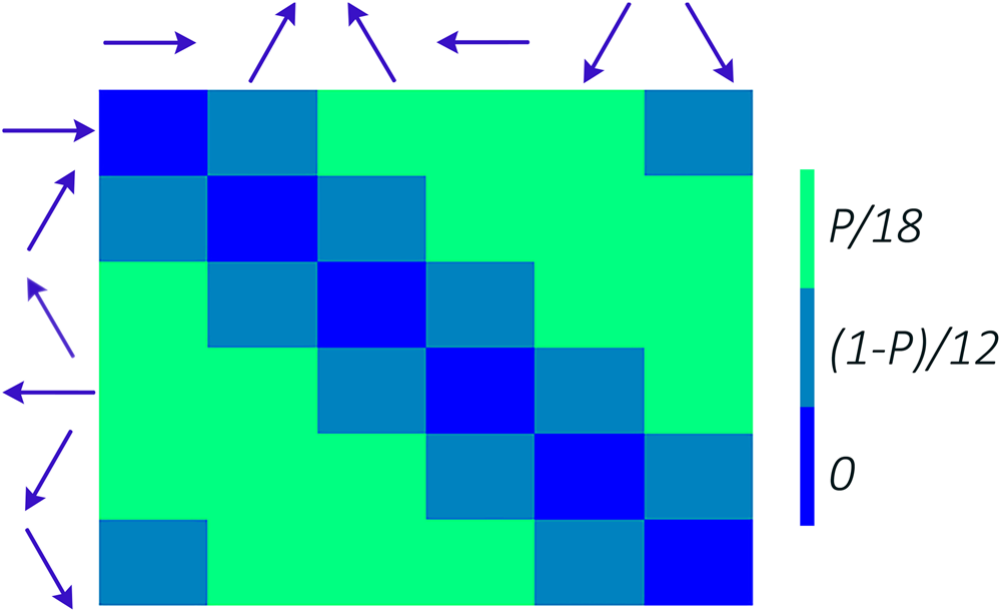
Rearranged invariant distribution of the transition matrix. The entries are stationary probability for transition between different directions. The blue arrows indicate the change in direction relative to the previous direction, e.g. the (1, 2) element of the diagram indicates the probability of a *π*/3 change in direction regardless of the current direction. Based on the observations^9^, we set *P*~1/7. Notably, disappearing of *α* and *β* (*α* + *β* = 1) on long times remarks that short memory of this Markov chain of second order cannot affect the future of the process on large scales.

Rearranged form of *ω*_0_ in colored matrix is depicted in Fig. 3. In this figure the blue arrows indicate the change in direction relative to the previous direction. Obviously, the probability of choosing each of six possible directions of *S* in a homogeneous environment is 1/6, that is why the summation of elements of *ω*_0_ both in a row and in a column gives 1/6. Another notable point in an invariant distribution is disappearing of *α* and *β* on longtimes (see Fig. 3).

**Figure 4:**
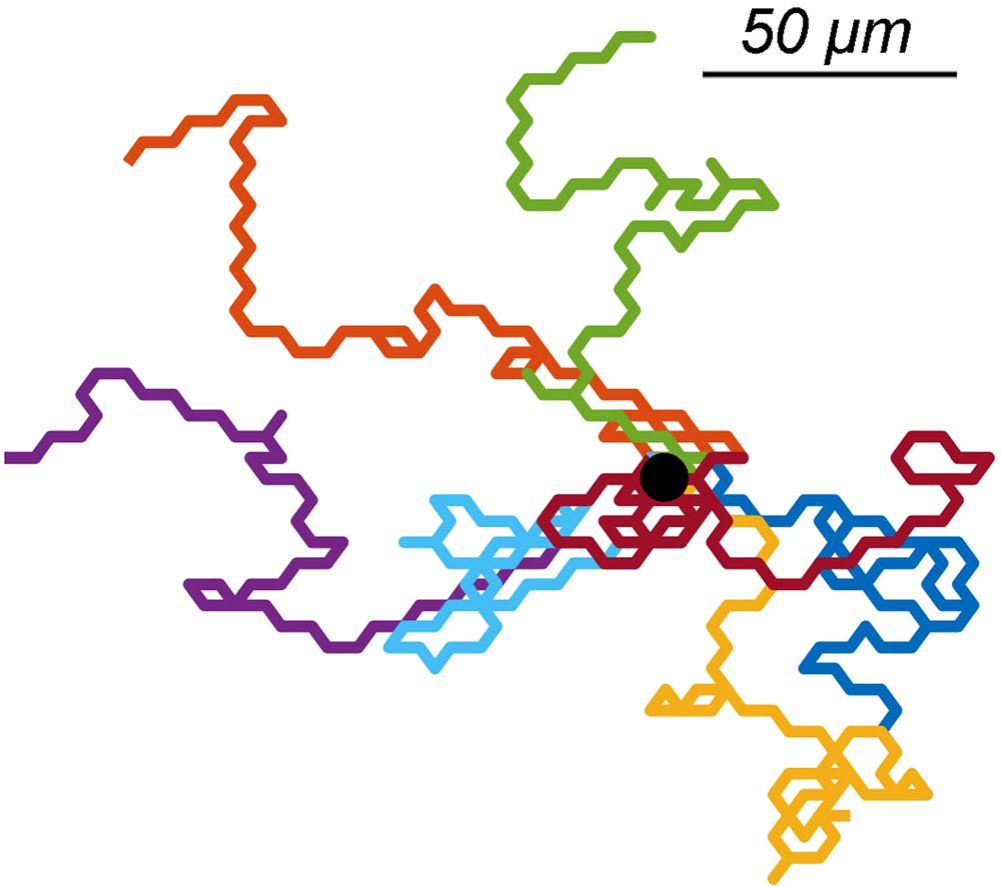
The trajectories of 7 cells during 15 min in a homogeneous medium. The black dot indicates the common origin of the trajectories.

As stated before, we assume that the persistence of cell movement is based on the ratio of splitting versus de novo pseudopodia. Thus, it is necessary to couple the cell’s centroid movement to the direction of growing pseudopodium. Let us assume that every extended pseudopodium leads to movement of the whole cell body and besides, the cell moves along the same direction of the extended pseudopodium. We define an orthonormal basis with the unit vectors $\hat{x}$ and $\hat{y}$ on the surface that the cells are crawling on it. The position of each cell is characterized in Cartesian coordinates by $\overrightarrow{r}=x\hat{x}+y\hat{y}$. In reality not all of the extended pseudopodia are followed by the cell body displacement. Indeed, only ~60% of these protrusions contribute to cell movement. But the size of proceeded length differs from step to step between ~0.25 *μm* to ~10 *μm*, depend on the previous state of the cell^9^. Here for simplicity, we assume that all the extended pseudopodia lead to the whole body displacement but with a fixed step size which is equal to 6 *μm*. The time resolution of the simulations, *i.e*. the time steps, are equal to 20 seconds. Figure 4 illustrates the trajectories of 7 independent tracers in the absence of chemical stimulants concentration gradients. As we expect spreading out of the cells on the surface is isotropic.

### Chemotactic Movement

**Figure 5:**
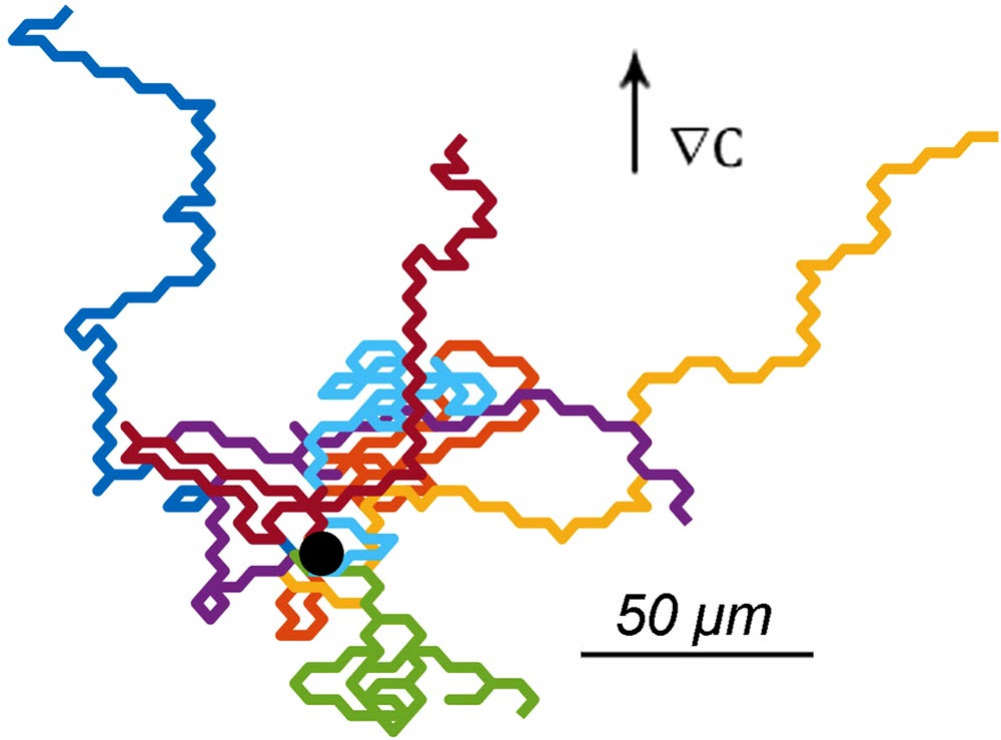
The tracks of 10 cells during 15 min in an inhomogeneous medium for which the coupling constant is equal to *ε* = 0.04. The black dot indicates the common initial point of the stochastic movement of the cells.

In the absence of chemical gradient, the system is entirely homogenous and isotropic. One of the consequences of this fact is that the corresponding transition matrix *P* ^(0)^ would be invariant under the index translation, *i.e*. ${P}_{i,j}^{\mathrm{(0)}}\to {P}_{i+\mathrm{1,}j+1}^{\mathrm{(0)}}$. However, in the presence of cAMP concentration gradient the index translation symmetry breaks down. Experiments show that even in such an inhomogeneous medium the pseudopodia do not bend towards the gradient and still are extended perpendicular to the local cell surface curvature^27^. But the gradient of cAMP induces a small bias in the direction of pseudopod extension, without significantly affecting parameters such as pseudopod frequency or size^29^. This fact suggests that the system still has the parity transformation, *i.e*. the mirror symmetry with respect to the gradient axis. Let us assume that the state of growing pseudopodia in the presence of external cues are still given by *S* in the relation (1). It is plausible to assume that the chemotactic movement of the cells are additive to their random movement. Thus the transition matrix between different states in the inhomogeneous medium *P*, is superposition of transition matrix in the homogeneous medium, *P* ^(0)^ and an additional matrix which corresponds to the cell’s attempt to get aligned with the preferred spatial direction, *i*.*e*. the gradient of cAMP. Clearly, the additional matrix has to depend on concentration gradient of the chemoattractant. Besides, every entry of this matrix should be sensitive to the angle between the prospective direction of growing pseudopodium and the gradient of cAMP. We opt the inner product between these two vectors as our theoretical counterpart in this case. As the parity symmetry implies, one should discard the probability of protruding along the opposite direction of the current direction at the cell’s posterior in the next step. The only restriction on the additional term is that the sum of each row should be zero. Clearly, with all the above characteristics the condition is going to automatically fulfill. Let us assume that $\hat{y}$ is the direction of the spatial gradient of cAMP and ${\hat{n}}_{j}$ represents the probable direction of expanding pseudopodium in the next step, then

9 $${P}_{ij}={P}_{ij}^{\mathrm{(0)}}+\varepsilon \,\hat{y}\cdot {\hat{n}}_{j},$$

where $\varepsilon =\varepsilon (C,\,\nabla C)$ is the function which gives the coupling between cell’s overall orientation and the gradient of chemoattractant and depends on both concentration of cAMP and its gradient. We refer it as the coupling coefficient. Dimensional analysis suggests that this function in this linear approximation would be equal to $\varepsilon =\gamma \tfrac{L\nabla C}{C}$, where *L* is the typical size of a cell (~10 *μm*) and *γ* is a numerical constant, called coupling parameter, that cannot be merely determined by the dimensional analysis method. Now one can find the stationary state for the cell’s chemotactic movement in this field. Owing to the size of the transition matrix *P* ^(0)^, finding an analytical solution for the stationary state of the system may be difficult, if possible. Here, applying perturbation methods, we solve the problem for the system in weak-coupling regime (see Supporting Information, Appendix B for details). As every individual probability, *P*_*ij*_, must be between 0 and 1, *i.e*. $\forall i,\,j\,0\le {P}_{ij}^{\mathrm{(0)}}+\varepsilon \,\hat{y}\cdot {\hat{n}}_{j}\le 1$, the maximum valid value for applying the perturbation method is *ε* = 0.043. By weak-coupling regime, we refer to the inhomogeneous fields of cAMP concentration for which *ε* ≤ 0.043. Figure 5 depicts the trajectories of 10 independent tracers in the presence of chemical stimulants concentration gradients, where the coupling coefficient between pseudopodium extension direction and gradient of external stimulant is equal to *ε* = 0.04. Clearly, there is an upward drift in line with the gradient of stimulant concentration.

## Results

**Figure 6:**
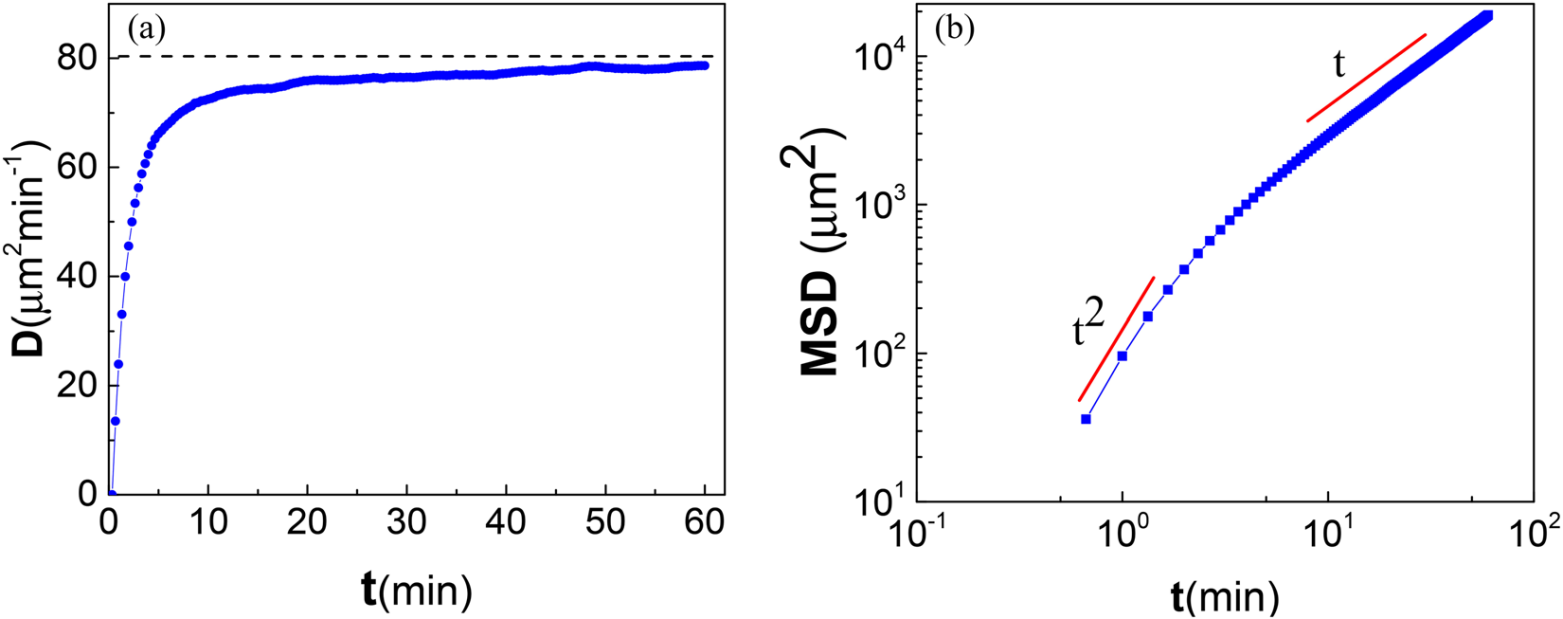
(**a**) Diffusion coefficient, *D* = 〈*r* ^2^(*t*)〉/4*t*, plotted as a function of *t*. For a random walk, diffusion coefficient would give rise to a line with zero slope on long times. (**b**) Log-log plot of the mean-squared displacement in respect to time. The transition between ballistic and diffusive regimes occurs after around 5 minutes. The number of realizations is *N* = 10000.

In Fig. 4 we show the trajectories of 7 independent tracers in a homogeneous medium. It is well-known^11^ that the relation

10 $$\langle {r}^{2}(t)\rangle =2{v}^{2}[\tau t-{\tau }^{2}\mathrm{(1}-{\exp }^{-t/\tau })]$$

governs the mean square displacement 〈*r* ^2^〉 of a random motion with persistence. Where *v* is a typical velocity of the tracer and *τ* is its persistence time. The mean squared displacement (MSD) is calculated as

11 $$MSD=\langle {(r-{r}_{0})}^{2}\rangle =\frac{1}{N}\,\sum _{n=1}^{N}\,{({r}_{n}(t)-{r}_{n}\mathrm{(0))}}^{2},$$

where *N* is the number of realizations to be averaged, *r*_*n*_(0) is the initial position of each tracer and *r*_*n*_(*t*) is the position of each individual in determined time *t*. Here, *N* = 10000 and the reference position of each cell is the origin *i*.*e*., *r*_*n*_(0) = 0. Figure 6(a) shows the plot of the function *D* = 〈*r* ^2^(*t*)〉/4*t* with respect to time, which give information of diffusive behavior in a typical 2–dimensional random walk process over time. On long times the function is equal to diffusion coefficient of the tracers. The graph shows a dramatic increase in the first 5 minutes and reaches approximately to a plateau near 80 *μm* ^2^/*min* within 30 minutes. In Fig. 6(b) the mean square displacement of the process in Log-Log scale has been illustrated. The crossover between the ballistic and diffusive regimes is clearly visible on the plot. Fitting the MSD to Eq. 10, yields the values *v* = 11.64 ± 0.25 *μm*/*min* and *τ* = 1.00 ± 0.35 *min* for the speed and the persistence time respectively, which is in close agreement with the experiment^9^.

**Figure 7:**
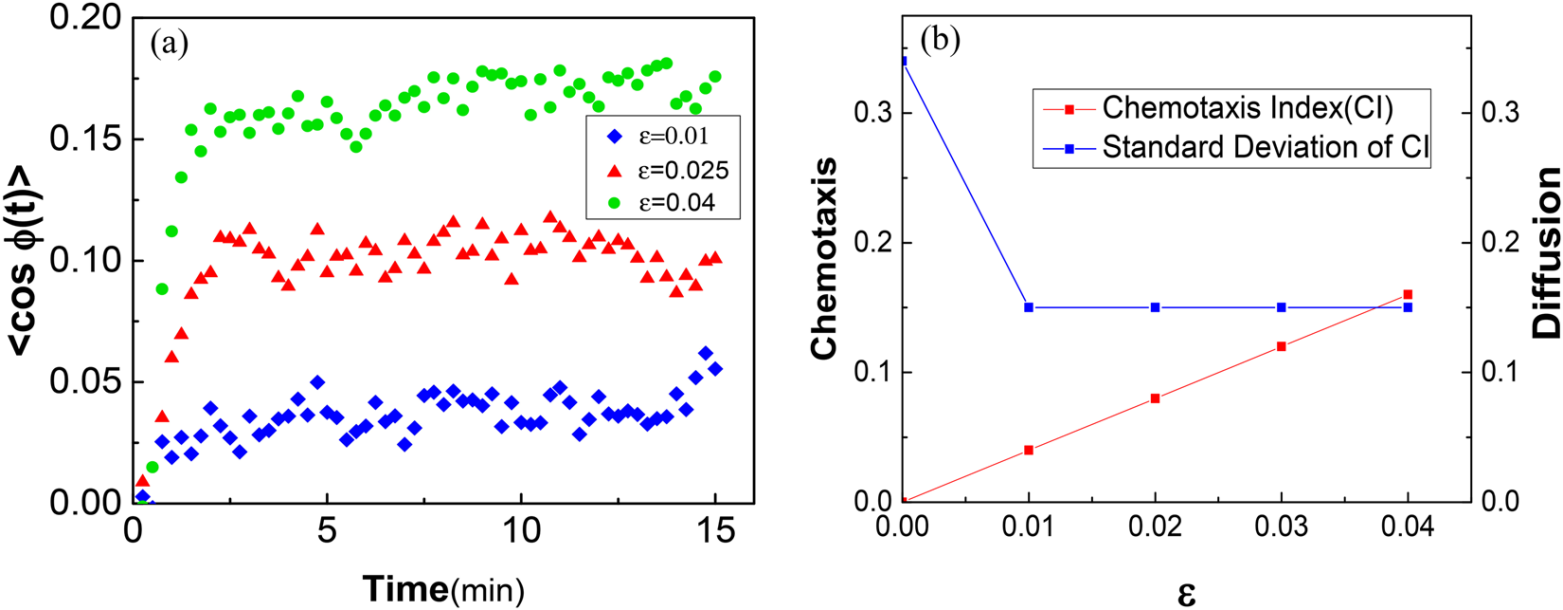
(**a**) 〈cos *ϕ*〉 plotted as a function of *t* for different values of coupling constants *ε*, *ϕ* is the angle between prospective pseudopodium and concentration gradient of cAMP. (**b**) Chemotaxis Index (CI) plotted as a function of the coupling coefficient *ε*. CI is a measure of how much a process does get biased in comparison with random motion. Still, the process includes randomness which can be indicated by the standard deviation of CI.

In the presence of constant gradient of chemoattractant cAMP, the cells start to co-orient their movements with the gradient direction. Let us assume that the angle between prospective pseudopodium and concentration gradient of cAMP is *ϕ*. The population average of this angle 〈cos *ϕ*(*t*)〉 is a proper quantity to measure the cell’s biased orientation in comparison with its random orientation in a homogeneous medium. As depicted in Fig. 7(a), even for a small value of coupling constant, there is a considerable tendency to move forward along the cAMP concentration gradient. Notably, 〈cos *ϕ*〉 reaches to a steady amount rather soon even for small values of *ε*. It is worth to mention that this amount is equal to zero in the homogeneous medium (See Fig. 3).

As an important illustration of the general features of chemotactic movement, we consider the Chemotaxis Index quantity, Fig. 7(b). Chemotaxis Index measures the chemotactic movement of a cell towards the chemical gradient with respect to its entire movement. One can calculate chemotactic index as the time average of 〈cos *ϕ*〉. It is apparent from the left-hand plot of Fig. 7(b) that chemotaxis index linearly increases with coupling constant for the allowed values of *ε*. As the range of *ε* considered here is low, thermal agitation still can affect the cell’s movement. To get a feeling of the influence of random thermal agitation one can measure the standard deviation of the chemotaxis index for different values of coupling constant, the right-hand plot of Fig. 7(b). Evidently, the chemotactic movement get comparable with diffusive propagation of the cells only near *ε* = 0.04. As a physical analogue, one may think of the low drift velocity of an ion induced by a weak electric field while the ion experiences countless collisions with fixed atoms in a circuit.

## Discussion

In recent years, analyzing the locomotion of various eukaryotic cells have been of interest^8–10,12,13,16–21^. All of these studies share this outcome that the experimental results deviate from the Ornstein-Uhlenbeck model of persistent random walk. Dieterich *et al*.^21^ report superdiffiusive behavior in the movement of the crawler cells. Therefore, they suggest that the fractional Klein-Kramers equation governs the cell’s motility. However, Li *et al*.^13^ did not observe any apparent intrinsic scale invariance in cell trajectories, which is the essential feature of a typical Lévy walk. There are also theoretical studies which shows that as far as the linear diffusion equation (even anomalous) meets both time- and space-translational invariance, like what the cells experience in homogeneous medium, the variance of movement is an at most linear function of time^30^. Hence, it seems that super-diffusive interpretation of the cell movement is a consequence of short duration of the related experiment and if one be patient enough, can meet the long time diffusive behavior of these cells. Hasstert^8^, Li *et al*.^13^ and Cooper *et al*.^31^ have addressed the same problem by presenting stochastic descriptions that reproduces the so- called zig-zag trajectories of the cells. Haastert^8^ proposes a model for the persistent random walk based on the observed angular frequencies of pseudopod extensions. By applying Monte Carlo simulations, he shows that the critical elements of this stochastic process are the ratio of persistent splitting pseudopodia relative to random de novo pseudopodia, the Left/Right alternation, the angle between pseudopodia and the variance of this angle. However, in his simulations, as the direction of the simulated de novo and splitting pseudopodia are random, there is always a limited probability that a simulated pseudopodium in de novo type would be recognized in experiments as splitting pseudopodium and vice versa.

Incorporating the principal elements of Haastert’s model^8^, we proposed a discrete model to revisit the question of directed motion of eukaryotic cells. Indeed, discretizing the possible angles along which one pseudopodium can extend, prevents shuffling of the simulated splitting and de novo pseudopodia. Based on our model, the cell selects its pseudopodium growth direction based on a second order Markov chain in the absence of external cues. Making the process second order, which is supported by the experiments^9^, has no effect on the stationary state of the cell’s migration. Indeed, the short memory of this process vanishes on the cell’s long time diffusive behavior (See Fig. 3). Nevertheless, it can postpone the cross over between ballistic and diffusive regimes (See Fig. 1). Thus, to describe the persistent behavior of the cells there is no need to attribute fat tail distributions to spatial steps.

Andrew *et al*.^7^ and Bosgraaf *et al*.^9^ define splitting pseudopods as pseudopods that originate from, or are formed in the domain of, an existing pseudopod. Thus, correct attribution of the membrane protrusions depends on defining the boundary between a pseudopod and the rest of the cell, and the correct experimental detection of this boundary. Based on experimental observation, the detected angle between two split-split pseudopodia has a bimodal distribution with peaks of about 55 degrees to the right or left relative to the previous pseudopod. However, distinction between splitting and de novo pseudopodia at the tails of this distribution is more challenging. Indeed, although cells shape change as they move, their shapes remain relatively smooth and the pseudopods grow perpendicular to the membrane. Therefore, a larger angular difference generally implies a larger spatial distance between the tips of two successive pseudopodia. That is, it is more likely that a fraction of de novo pseudopods have been detected as splitting pseudopods at this region. Hence, the tails of the distribution may be lighter than it is. To build the space of state, we assume that splitting at the side of previous pseudopodium occurs at discrete angles - with approximately *π*/3 radians turn- in the anterior part of the current active area of cell. Apparently, the main part of simplification has been applied in considering discrete states for extending de novo pseudopodia. However, as this type of pseudopodia rarely occur (growing a splitting pseudopodium is seven times more probable than a de novo pseudopodium^9^), one may consider this simplification something like a mean-field approximation. The statistical features of the model for the cell’s centroid motility are in well agreement with the corresponding measured experimental and also simulated quantities (with considering all the complexities of non-discrete states). This may suggest that the model can describe the locomotion of the wild-type cells with statistically regular shape fairly well. The reported speed and persistence time for a typical wild type cell during 15 minutes are *v* = 10.4 ± 2.1 *μm*/*min* and *τ* = 3.4 ± 0.5 *min*, respectively^9^. By applying our discrete model, one obtains *v* = 11.64 ± 0.25 *μm*/*min* and *τ* = 1.00 ± 0.35 *min* for these quantities along one hour of simulation. It is seen that within the error bars the measured speed matches well with the experiment. However, the predicted persistence time somehow differs from that of experiment. A possible explanation for this might be that these tracers have to alter their direction at least *π*/3 in two successive steps. The discrete model provides a framework to develop a suitable computational schemes which is generalizable to chemotactic movement as well.

In the presence of external stimulants, the cell tends to align itself with the gradient of the chemoattractant cAMP^7,10^. This suggests that the tendency and random locomotion of the cell “add up” to propel the cell toward the gradient. In this case both the absolute value of chemoattractant concentration and its gradient incorporate to motivate the cells to crawl through an inhomogeneous field at the same time. In most of the previous studies, these processes has been dealt separately^10,16,17^. Haastert^10^ extracts the probabilities as well as the gradient induced bias in chemotactic motion from experiments and performs Monte Carlo simulations to quantify Chemotaxis index of a population of individuals with respect to cAMP steepness in the environment. His measured Chemotaxis index is in close agreement with experimental data, however as the model for chemotaxis is firmly based on the ordered extension of pseudopodia in the absence of chemoattractants, the above-mentioned difficulties with Monte Carlo simulation are still relevant. Our model is also built on pseudopod extension-based description which is previously developed by Haastert^10^, but with fewer parameters. We also include the dependency of the chemotactic response to the gradient of cAMP and its local concentration in our model. Previously, this sensitivity has been investigated in some experiments^32,33^. By applying information theory techniques, Fuller *et al*.^32^ show that for shallow gradients and small local concentrations, the extra cellular fluctuations limit the chemotactic response. In addition, for steep gradients and high local concentrations the observed chemotactic response is lower than what one may predict from a simple ligand-receptor binding process. Inspiring by these observations, we assume that the chemotactic response of the cell to its chemical environment is proportional to the ratio of the steepness $\nabla C$ and absolute value of concentration field *C* (See Eq. 9 and its underneath statement). The parameter with which this proportionality get adjusted is coupling parameter *γ*. This parameter is a phenomenological variable that have to measure experimentally. Indeed, Eq. 9 suggests a practical procedure to determine coupling coefficient *ε* values from the transition probabilities quantified from the corresponding experiments for variant pairs of *C* and $\nabla C$. This is a fundamental issue for future research which requires carrying out more experiments. Investigating the dynamical properties of the cells in weak-coupling regime in a broader sense might pave our way toward gaining a better insight of systems in which responsiveness of the cells to the external field is weak, *e.g*. in the ‘back of the wave’ problem.

Here, taking the advantage of perturbation methods, we have studied the cell’s migration in weak coupling regime, where *ε* ≤ 0.043. The approach is not extendable to the strong coupling regime where the coupling coefficient *ε* is higher. Thus, developing new approaches to study this regime needs more effort and is beyond the aim of this paper.

In summary, we have studied the amoeboid locomotion and chemotaxis in *Dictyostelium discoideum* by tracking pseudopodium growth direction. We have applied Markov chain of second order to describe the behavior of eukaryotic locomotion in homogenous medium. According to the model, short memory of the second order Markov chain vanishes on the cell’s stationary state and hence leads to a smooth transition from persistent random walk to purely diffusive behavior for squared displacement. The chemotactic response of the cell in inhomogeneous medium depends both on the local background concentration and its gradient. Coupling coefficient *ε* measures this dependency for different possible pairs of them. Utilizing perturbation method, we showed that for the cells which undergo chemotaxis under influence of weak coupling, there is a linear dependence between chemotaxis index and coupling coefficient *ε*. Our method might help shed light on dealing with the phenomenon of cell aggregation from a theoretical point of view.

## Electronic supplementary material

Discrete Modeling of Amoeboid Locomotion and Chemotaxis in Dictyostelium discoideum by Tracking Pseudopodium Growth Direction
