# Supplementary material for: Discrete Modeling of Amoeboid Locomotion and Chemotaxis in Dictyostelium discoideum by Tracking Pseudopodium Growth Direction
Source: Sci Rep. 2017 Oct 4;7:12675. doi: 10.1038/s41598-017-12656-1 (PMC5627298; doi:10.1038/s41598-017-12656-1)
Supplement: Supplementary file 1 — Discrete Modeling of Amoeboid Locomotion and Chemotaxis in Dictyostelium discoideum by Tracking Pseudopodium Growth Direction [file 41598_2017_12656_MOESM1_ESM.pdf]

Discrete Modeling of Amoeboid Locomotion and Chemotaxis in *Dictyostelium discoideum* by Tracking Pseudopodium Growth Direction

Zahra Eidi[1,*]

[1]Department of Physics, Institute for Advanced Studies in Basic Sciences (IASBS), Zanjan 45137-66731, Iran
[*]z.eidi@iasbs.ac.ir

ABSTRACT

Supplementary material presents more detailed information on the various issues discussed in the main text. The first section, Appendix A, illustrates all the entries of the transition matrix $P^{(0)}$. The second section, Appendix B, describes the perturbation method with which one can find the stationary state (invariant distribution) of the cells motility in the inhomogeneous medium.

Appendix A

By inserting the corresponding transition probabilities, the transition matrix $P^{(0)}$ reads as,

$$
P^{(0)} = \begin{array}{c} \\ (1,2) \\ (1,3) \\ (1,4) \\ (1,5) \\ (1,6) \\ \vdots \\ (6,1) \\ (6,2) \\ (6,3) \\ (6,4) \\ (6,5) \end{array}
\begin{array}{c}
\begin{array}{cccccccccccc} (1,2) & (1,3) & (1,4) & (1,5) & (1,6) & \cdots & (6,1) & (6,2) & (6,3) & (6,4) & (6,5) \end{array} \\
\left(\begin{array}{cccccccccccc}
0 & 0 & 0 & 0 & 0 & \cdots & \alpha(1-p) & 0 & 0 & 0 & 0 \\
0 & 0 & 0 & 0 & 0 & \cdots & (1-p)/2 & 0 & 0 & 0 & 0 \\
0 & 0 & 0 & 0 & 0 & \cdots & (1-p)/2 & 0 & 0 & 0 & 0 \\
0 & 0 & 0 & 0 & 0 & \cdots & (1-p)/2 & 0 & 0 & 0 & 0 \\
0 & 0 & 0 & 0 & 0 & \cdots & \beta(1-p) & 0 & 0 & 0 & 0 \\
\vdots & \vdots & \vdots & \vdots & \vdots & \cdots & \vdots & \vdots & \vdots & \vdots & \\
\beta(1-p) & 0 & 0 & 0 & 0 & \cdots & 0 & 0 & 0 & 0 & 0 \\
(1-p)/2 & 0 & 0 & 0 & 0 & \cdots & 0 & 0 & 0 & 0 & 0 \\
(1-p)/2 & 0 & 0 & 0 & 0 & \cdots & 0 & 0 & 0 & 0 & 0 \\
(1-p)/2 & 0 & 0 & 0 & 0 & \cdots & 0 & 0 & 0 & 0 & 0 \\
\alpha(1-p) & 0 & 0 & 0 & 0 & \cdots & 0 & 0 & 0 & 0 & 0
\end{array} \right)_{30\times 30}
\end{array}
$$

Here, the horizontal and vertical values stand for X_{t+1} and X_t, respectively. One can distinguish 36 blocks, each of which is 5×5, in this matrix as follows:

$$
P^{(0)} = \begin{pmatrix}
\overline{\mathbb{O}} & A & B & B & B & C \\
D & \overline{\mathbb{O}} & E & F & F & F \\
G & H & \overline{\mathbb{O}} & I & G & G \\
J & J & K & \overline{\mathbb{O}} & L & J \\
M & M & M & N & \overline{\mathbb{O}} & O \\
P & Q & Q & Q & R & \overline{\mathbb{O}}
\end{pmatrix} \qquad (1)
$$

$$
A = \begin{pmatrix}
\beta(1-p) & 0 & 0 & 0 & 0 \\
(1-p)/2 & 0 & 0 & 0 & 0 \\
(1-p)/2 & 0 & 0 & 0 & 0 \\
(1-p)/2 & 0 & 0 & 0 & 0 \\
\alpha(1-p) & 0 & 0 & 0 & 0
\end{pmatrix}, B = \begin{pmatrix}
p/3 & 0 & 0 & 0 & 0 \\
p/3 & 0 & 0 & 0 & 0 \\
p/3 & 0 & 0 & 0 & 0 \\
p/3 & 0 & 0 & 0 & 0 \\
p/3 & 0 & 0 & 0 & 0
\end{pmatrix}, C = \begin{pmatrix}
\alpha(1-p) & 0 & 0 & 0 & 0 \\
(1-p)/2 & 0 & 0 & 0 & 0 \\
(1-p)/2 & 0 & 0 & 0 & 0 \\
(1-p)/2 & 0 & 0 & 0 & 0 \\
\beta(1-p) & 0 & 0 & 0 & 0
\end{pmatrix}
$$

$$
D = \begin{pmatrix}
\beta(1-p) & 0 & 0 & 0 & 0 \\
\alpha(1-p) & 0 & 0 & 0 & 0 \\
(1-p)/2 & 0 & 0 & 0 & 0 \\
(1-p)/2 & 0 & 0 & 0 & 0 \\
(1-p)/2 & 0 & 0 & 0 & 0
\end{pmatrix}, E = \begin{pmatrix}
\alpha(1-p) & 0 & 0 & 0 & 0 \\
\beta(1-p) & 0 & 0 & 0 & 0 \\
(1-p)/2 & 0 & 0 & 0 & 0 \\
(1-p)/2 & 0 & 0 & 0 & 0 \\
(1-p)/2 & 0 & 0 & 0 & 0
\end{pmatrix}, F = \begin{pmatrix}
p/3 & 0 & 0 & 0 & 0 \\
p/3 & 0 & 0 & 0 & 0 \\
p/3 & 0 & 0 & 0 & 0 \\
p/3 & 0 & 0 & 0 & 0 \\
p/3 & 0 & 0 & 0 & 0
\end{pmatrix}
$$

$$
G = \begin{pmatrix}
0 & p/3 & 0 & 0 & 0 \\
0 & p/3 & 0 & 0 & 0 \\
0 & p/3 & 0 & 0 & 0 \\
0 & p/3 & 0 & 0 & 0 \\
0 & p/3 & 0 & 0 & 0
\end{pmatrix}, H = \begin{pmatrix}
0 & (1-p)/2 & 0 & 0 & 0 \\
0 & \beta(1-p) & 0 & 0 & 0 \\
0 & \alpha(1-p) & 0 & 0 & 0 \\
0 & (1-p)/2 & 0 & 0 & 0 \\
0 & (1-p)/2 & 0 & 0 & 0
\end{pmatrix}, I = \begin{pmatrix}
0 & (1-p)/2 & 0 & 0 & 0 \\
0 & \alpha(1-p) & 0 & 0 & 0 \\
0 & \beta(1-p) & 0 & 0 & 0 \\
0 & (1-p)/2 & 0 & 0 & 0 \\
0 & (1-p)/2 & 0 & 0 & 0
\end{pmatrix}
$$

$$
J = \begin{pmatrix}
0 & 0 & p/3 & 0 & 0 \\
0 & 0 & p/3 & 0 & 0 \\
0 & 0 & p/3 & 0 & 0 \\
0 & 0 & p/3 & 0 & 0 \\
0 & 0 & p/3 & 0 & 0
\end{pmatrix}, K = \begin{pmatrix}
0 & 0 & (1-p)/2 & 0 & 0 \\
0 & 0 & (1-p)/2 & 0 & 0 \\
0 & 0 & \beta(1-p) & 0 & 0 \\
0 & 0 & \alpha(1-p) & 0 & 0 \\
0 & 0 & (1-p)/2 & 0 & 0
\end{pmatrix}, L = \begin{pmatrix}
0 & 0 & (1-p)/2 & 0 & 0 \\
0 & 0 & (1-p)/2 & 0 & 0 \\
0 & 0 & \alpha(1-p) & 0 & 0 \\
0 & 0 & \beta(1-p) & 0 & 0 \\
0 & 0 & (1-p)/2 & 0 & 0
\end{pmatrix}
$$

$$M=\begin{pmatrix}0&0&0&p/3&0\\0&0&0&p/3&0\\0&0&0&p/3&0\\0&0&0&p/3&0\\0&0&0&p/3&0\end{pmatrix},N=\begin{pmatrix}0&0&0&(1-p)/2&0\\0&0&0&(1-p)/2&0\\0&0&0&(1-p)/2&0\\0&0&0&\beta(1-p)&0\\0&0&0&\alpha(1-p)&0\end{pmatrix},O=\begin{pmatrix}0&0&0&(1-p)/2&0\\0&0&0&(1-p)/2&0\\0&0&0&(1-p)/2&0\\0&0&0&\alpha(1-p)&0\\0&0&0&\beta(1-p)&0\end{pmatrix}$$

$$P=\begin{pmatrix}0&0&0&0&\beta(1-p)\\0&0&0&0&(1-p)/2\\0&0&0&0&(1-p)/2\\0&0&0&0&(1-p)/2\\0&0&0&0&\alpha(1-p)\end{pmatrix},Q=\begin{pmatrix}0&0&0&0&p/3\\0&0&0&0&p/3\\0&0&0&0&p/3\\0&0&0&0&p/3\\0&0&0&0&p/3\end{pmatrix},R=\begin{pmatrix}0&0&0&0&\alpha(1-p)\\0&0&0&0&(1-p)/2\\0&0&0&0&(1-p)/2\\0&0&0&0&(1-p)/2\\0&0&0&0&\beta(1-p)\end{pmatrix}$$

Appendix B

Let us assume that P is the perturbed evolution operator

$$P=P^{(0)}+\varepsilon P^{(1)} \tag{2}$$

where all its entries are non-negative and in addition $s=\mathbf{1}$ is its trivial right eigenvector corresponding to eigenvalue 1. The unperturbed evolution operator $P^{(0)}$ satisfies similar criteria. The aim is to obtain ω, the left eigenvector of P corresponding to the eigenvalue 1.

$$\omega P=\omega \tag{3}$$

Let us assume further that the eigenvalue 1 of $P^{(0)}$ is nondegenerate, so there is only one left eigenvector ω_0 satisfying

$$\omega_0 P^{(0)}=\omega_0 \tag{4}$$

Besides, let us suppose that $\omega=\omega_0+\varepsilon\omega_1$. One can substitute Eq.4 in Eq.2 , keep the relation up to the first order of ε and find

$$\omega_0 P^{(1)}=\omega_1(1-P^{(0)}), \tag{5}$$

Clearly, the relation is not invertible since ω_0 is in the kernel of $(1-P^{(0)})$. But, Eq.5 does in fact have one and only one solution for ω_1 (assuming that the generalized eigenvectors of P corresponding to one are nondegenerate). The reason is that although $(1-P^{(0)})$ is not invertible, the left-hand side of Eq.5 is in the image of $(1-P^{(0)})$, as the image of $(1-P^{(0)})$ is the kernel of s, and the left-hand side is killed by s. This proves the existence of a solution for ω_1. The uniqueness comes from the fact that any ω_1 is in the kernel of s, and the restriction of $(1-P^{(0)})$ to the kernel of s is one-to-one.
To find the solution, it is enough to discard the last row of the matrices, and subtract the last column from other columns to obtain the matrices with one less row and column. Let us consider the covectors E^i which along with ω_0 form a basis, and the vectors E_j which along with s form a basis, satisfying the following relation.

$$E^j E_i=\delta_i^j \tag{6}$$

A simple choice would be

$$(E_i)^j=\delta_i^j, \tag{7}$$
$$(E_i)^n=0, \tag{8}$$
$$(E^i)_j=\delta_j^i, \tag{9}$$
$$(E^i)_n=0. \tag{10}$$

These are in fact the simple basis of the space of states and its dual, apart from the n'th vector. Let us define the covector v such that all its elements are zero except the last one which is -1. Now,

$$e^i = E^i - v \tag{11}$$

The set e^i's along with ω_0 is a basis for the space of the states. So, one can expand ω_1 as

$$\omega_1 = (\omega_1)_i e^i + a\omega_0 \tag{12}$$

But a is zero as s kills both ω_1 and e^i's but not ω_0. So, Eq.5 becomes

$$(\omega_1)_i e^i (1 - P^{(0)}) = \omega_0 P^{(1)} \tag{13}$$

Hence,

$$(\omega_1)_i e^i (1 - P^{(0)}) E_j = \omega_0 P^{(1)} E_j \tag{14}$$

Defining the $(n-1) \times (n-1)$ matrix N through

$$N^i_j = e^i (1 - P^{(0)}) E_j, \tag{15}$$

it is seen that

$$(\omega_1)_i = (N^{-1})^i_j \omega_0 P^{(1)} E_j, \tag{16}$$

$$\omega_1 = e^i (N^{-1})^i_j \omega_0 P^{(1)} E_j. \tag{17}$$

The normalization for ω_1 is that the sum of its elements to be equall to zero, and that's automatically guaranteed. The solution would be simpler if e_i's and e^i's were eigenvectors. In that case,

$$\omega_1 = \Sigma_i \frac{e^i \omega_0 P^{(1)}}{1 - \lambda_i} E_i, \tag{18}$$

Where

$$e^i P^{(0)} = \lambda_i e^i. \tag{19}$$

But for the finite dimensional matrices inverting N is much simpler than finding all of the eigenvectors of $P^{(0)}$.
